# Effective Removal of Chromium(III) from Low Concentration Aqueous Solution Using a Novel Diazene/Methoxy-Laced Coordination Polymer

**DOI:** 10.3390/polym9070273

**Published:** 2017-07-09

**Authors:** Lei-Lei Liu, Yun Xing, Hui-Ying Yu, Cai-Wen Zhang, Meng-Qi Ye, Ming-Zhen Miao, Cai-Xia Yu

**Affiliations:** Henan Key Laboratory of New Optoelectronic Functional Materials, College of Chemistry and Chemical Engineering, Anyang Normal University, Anyang 455000, China; lvyh086@aynu.edu.cn (Y.X.); H1971145219@163.com (H.-Y.Y.); 17839164412@163.com (C.-W.Z.); 15690611661@163.com (M.-Q.Y.); mmz1994a@163.com (M.-Z.M.)

**Keywords:** diazene/methoxy-laced coordination polymer, chromium(III) removal, adsorption, low concentration, selectivity

## Abstract

In this study, a novel coordination polymer [CdL_2_(H_2_O)_0.5_]*_n_* (**1**), [HL = 4-(2-(4-((pyridin-3-yl)methoxy)phenyl)diazenyl)benzoic acid] was fabricated via an in situ ligand transformation reaction under solvothermal conditions. The as-prepared polymer exhibited a selectivity and efficiency for Cr(III) removal with a high uptake capacity of 106.13 mg·g^−1^. Interestingly, even in the low concentration (0.02–0.20 ppm), it still performs a relatively high efficiency (≥ 92.5%) towards the removal of Cr(III) in aqueous solution. Remarkably, it also presents good selectivity and high efficiency (93.3%) for Cr(III) removal in the presences of interfering metal ions. The good removal performance for Cr(III) was demonstrated to be a structure-dependent chemical process between polymer and Cr(III) involving the diazene and methoxy groups in polymer **1**, which happened not only on the surfaces of the adsorbent but also in the pores of polymer, giving rise to a strong affinity toward Cr(III) adsorption. The possible adsorption mechanism of Cr(III) was proposed and systematically verified by FT-IR, scanning electron microscope (SEM), atomic force microscope (AFM) and energy dispersive spectrometer (EDS) measurements.

## 1. Introduction

Heavy metal pollution has become a serious threat to environment and public health, since they are highly toxic and accumulate throughout the food chain [1,2]. Chromium is a prevalent and highly reactive pollutant which dissipates into the ecosystems from a variety of industrial activities such as electroplating, leather tanning, mining, textile dyeing, wood preserving, chromate preparation and metal finishing [3]. Although trace amounts of chromium are required for biological functions, excess is reported to cause health-related problems like rashes, ulcers, respiratory disorders, cardiovascular diseases and weakening of immune systems [4,5].

In aqueous solution, chromium exists in two stable oxidation states: hexavalent chromium [Cr(VI)] and trivalent chromium [Cr(III)] [6]. Out of the two states, Cr(VI) is generally considered as more toxic because of its ability to cross cellular membranes easily and its strong oxidizing power [7]. However, Cr(III) can be as toxic as, or more toxic than Cr(VI) to freshwater algae, aquatic mosses, yeasts and fishes according to studies published after 2000 [8,9]. Suwalsky et al. demonstrated that Cr(III) cause more structural perturbation in human erythrocyte membrane than Cr(VI) [10]. Moreover, under prolonged or severe exposure to Cr(III), it can induce oxidative DNA damage, causing cancer [11,12]. Nevertheless, the researchers have paid more attention to the removal of Cr(VI) [13,14,15,16,17,18], but materials for the removal of Cr(III) were rarely reported [19,20,21,22].

At present, a wide range of treatment technologies, such as chemical precipitation, ion-exchange, adsorption, membrane separation, reduction, solvent extraction and reverse osmosis, have been developed to remove chromium from aqueous solution [13,14,15,16,17,18,19,20,21,22,23,24,25]. Most of them are effective at removing chromium from aqueous solution containing relatively high initial chromium concentrations [26]. However, the chromium removal efficiency of these technologies is still not satisfactory, such as a long detention time, poor selectivity of chromium and the higher residual concentration. According to the World Health Organization drinking water guidelines, the maximum allowable limit for chromium is 0.05 ppm [27]. As a consequence, developing more efficient Cr(III) removal materials, especially aiming at low-concentration Cr(III) systems, is significant for human health and environment.

Coordination polymers (CPs), constructed by the assembly of metal-containing units with appropriate organic linking groups, are a class of crystalline materials [28]. The design and synthesis of CPs have attracted an upsurge in research interest because of their tremendous potential applications in storage/adsorption, catalysis, separation, and drug delivery [29,30,31,32,33,34,35]. The ultimate aim of coordination chemistry is to control the structures of target products and investigate the relationships between structures and properties [29,36]. As is known to all, the selection of appropriate ligands is an important factor to rational design and synthesis of CPs with desired structures and properties, because they are highly tunable; even a small change can result in a remarkable diversity of both architectures and properties [37]. Recently, researchers started to construct functionalized CPs based on various types of ligands, which displayed good performance for effective removal of heavy metals in aqueous environments [38,39,40,41,42,43,44,45,46,47,48]. For example, Xu and co-workers reported the Zn/Zr-based CPs construction by carboxyl/thiols or carboxyl/thiol-ethers combinations, which showed good performance for Hg(II) removal from ethanol or aqueous solution [49,50]. Luo investigated the Zn-based CPs assembly from the carboxyl/hydroxyl and pyridyl/acylamide ligands, which exhibited the capture ability towards Hg(II) and UO_2_(I) ions in aqueous solution [51,52]. Morsali employed azine-decorated Zn(II) CPs as the sorbent to remove some heavy metal ions [Cd(II), Co(II), Cr(III), Cu(II) and Pb(II)] in aqueous solution [53,54]. In these CPs, the thiol, thiol-ether, hydroxyl, acylamide and azine groups did not coordinate with the metal ions in the self-assembly processes, but worked as the hands to capture the heavy metal ions. 

**Scheme 1:**
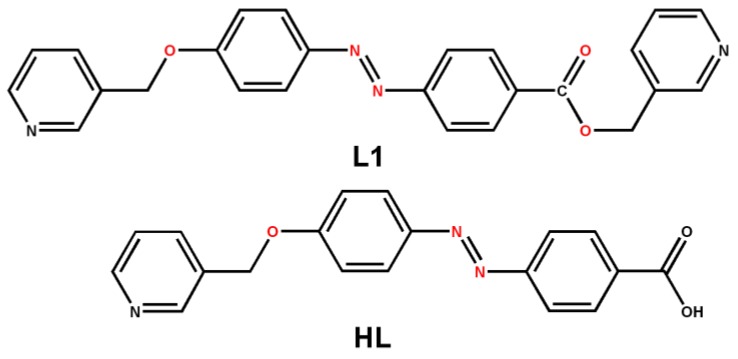
Chemical structures of the L and HL ligands.

Nowadays, the exploration of CPs for chromium removal from other metal ions, especially for selective removal of Cr(III) at low concentration, remains to be a challenge. Hence, in this article, a novel Cd(II)-based CP, [CdL_2_(H_2_O)_0.5_]*_n_* (**1**), was synthesized by Cd(OAc)_2_·2H_2_O and the designed L1 ligand with the diazene/methoxy/carbomethoxy laced groups (Scheme 1) and employed as the sorbent, which demonstrates a high uptake capability (106.13 mg·g^−1^) for Cr(III). It is noteworthy that it exhibits a good efficiency towards the removal of Cr(III) (97.7%), even in the magnitude of 0.02 ppm.

## 2. Materials and Methods 

### 2.1. Materials and General Methods

(pyridin-3-yl)methyl 4-(2-(4-((pyridin-3-yl)methoxy)phenyl)diazenyl)benzoate (L1) was prepared according to the literature method [55]. All other chemicals and reagents were obtained from commercial sources and used as received. Infrared (IR) spectra were recorded with a Varian 800 FT-IR spectrometer (Varian, Inc., Palo Alto, USA) (4000–400 cm^−1^). Powder X-ray diffraction (PXRD) was performed using a PANalytical X’Pert PRO MPD system (PANalytical B.V., Almelo, Holland).) (PW3040/60). Field emission scanning electron micrographs (SEM) were obtained with a JSM 6701F microscope (Japan Electronics Co., Ltd, Tokyo, Japan). Atomic force microscopy (AFM) images were obtained on a dimension edge microscope (Bruker Nano Inc., Santa Barbara, CA, USA) equipped with a tapping mode. Thermal analyses were performed with a Netzsch STA-449F3 thermogravimetric analyzer (Netzsch, Co., Selb, Germany) at a heating rate of 10 °C min^−1^ and a flow rate of 20 cm^3^·min^−1^ (N_2_). Simultaneous inductively coupled plasma optical emission spectrometry (ICP-OES) on a PerkinElmer Optima 8000 instrument (PerkinElmer Inc., Waltham, USA) was used for simultaneous determination of the target elements.

### 2.2. Preparation of [CdL_2_(H_2_O)_0.5_]_n_ (**1**)

A mixture of Cd(OAc)_2_·2H_2_O (11 mg, 0.04 mmol), L1 ligand (8 mg, 0.02 mmol), 1,4-benzenedicarboxylic acid (3 mg, 0.02 mmol), and 4 mL of MeCN/H_2_O (1:1 V/V) was sealed in a 10 mL Pyrex glass tube and heated at 170 °C for 3 days, then cooled to room temperature at a rate of 5 °C h^−1^. The orange blocks of **1** were collected and washed thoroughly with MeCN and dried in air. Yield: 3 mg (38%, based on L1). Anal. Calcd. for C_38_H_29_N_6_CdO_6.5_: C, 58.06; H, 3.72; N, 10.69. Found: C, 58.51; H, 3.25; N, 3.99. IR (KBr disc): 3263 (w), 2919 (w), 2897 (w), 1587 (s), 1537 (s), 1495 (s), 1428 (m), 1383 (s), 1297 (m), 1231 (s), 1190 (m), 1141 (m), 1103 (m), 1054 (m), 1017 (m), 871 (w), 835 (s), 784 (s), 696 (s), 645 (m), 550 (m), 517 (m), 421 (m)·cm^−1^. 

### 2.3. X-ray Data Collection and Structure Determination

Single crystals of **1** were obtained directly from the above preparations. All measurements were made on a Bruker Smart Apex-II CCD area detector (Bruker AXS, Karlsruhe, Germany) by using graphite monochromated Mo Kα (*λ* = 0.071073 nm). Its crystal was mounted on glass fibers at 296 K. Cell parameter was refined by using the program Bruker SAINT (Bruker AXS, Karlsruhe, Germany). The collected data was reduced by using the program Bruker *SAINT* A, and the absorption correction (multi-scan) was applied. The reflection data was also corrected for Lorentz and polarization effects. The crystal structure of **1** was solved by direct method refined on *F*^2^ by full-matrix least-squares technique with the SHELXTL-97 program (University of Göettingen, Göettingen, Germany) [56]. All H atoms in **1** were placed in geometrically idealized positions and constrained to ride on their parent atoms. A summary of the key crystallographic information for **1** is tabulated in Table S1. Crystallographic data have been submitted to the Cambridge Structural Database with deposition number CCDC 1439364.

### 2.4. Adsorption Test

The Cr(III) stock solution was prepared by dissolving CrCl_3_·6H_2_O in deionized water. All adsorption tests were carried out at 25 °C using 7 mg of polymer **1** and 100 mL Cr(III) stock solution. To analyze the effect of pH, the sorption tests were conducted under different pH conditions (3.0–6.0) adjusted using 0.1 mol·L^−1^ HCl or 0.1 mol·L^−1^ NaOH solutions. An adsorbent (7 mg) was added to 100 mL of 0.1 ppm Cr(III) solution under continuous stirring at 25 °C for 15 min.

For kinetic studies, the pH was fixed at the obtained optimum value. A typical adsorption experiment was performed by adding 7 mg of adsorbent into 100 mL of an 1 ppm Cr(III) solution under continuous stirring. Analytical samples were taken from the mixture solution at given time intervals and immediately separated by centrifugation. The residual concentration of Cr(III) in the solution was measured by ICP-OES spectrometer.

In order to obtain the adsorption isotherms of the Cr(III), solutions with varying initial concentrations of Cr(III) from 1 ppm to 100 ppm were treated with the same procedure as above to attain the equilibrium state. The amount of Cr(III) adsorbed at equilibrium *q_e_* (mg·g^−1^) and the removal efficiency were obtained from the following Equations:

(1) $$q_{e}=\frac{\left(C_{0}-C_{e}\right)V}{m}$$

(2) $$\mathrm{Removal}\;\mathrm{efficiency}\;(\%)=\frac{C_{0}-C_{e}}{C_{0}}\times 100\%$$

where *C*_0_ and *C*_e_ (mg·L^−1^) are the initial and equilibrium concentrations of Cr(III) in the solution, respectively. *V* (L) is the volume of the solution, and *m* (g) is the mass of the adsorbent.

The selectivity towards Cr(III) removal was valued by competitive adsorption in the presence of various competitive metal ions. A mixture of Cr(III), Na(I), Mg(II), K(I), Ca(II), Mn(II), Co(II), Ni(II), Cu(II) and Zn(II) was added into deionized water to obtain an initial concentration of 0.1 ppm for each ions. After a competitive adsorption equilibrium was reached, the concentrations of Cr(III), Na(I), Mg(II), K(I), Ca(II), Mn(II), Co(II), Ni(II), Cu(II) and Zn(II) in the remaining samples were detected using a ICP-OES spectrometer. The following Equations were used to evaluate the selectivity of polymer **1**. Static distribution coefficient:

(3) $$K_{d}=\frac{q_{e}}{C_{e}}$$

where *q*_e_ (mg·g^−1^) represents the adsorption capacity and *C*_e_ (mg·L^−1^) is the equilibrium concentration. 

## 3. Results and Discussion

### 3.1. Synthetic and Structural Characterization

As shown in Scheme 1, L1 ligand contains two rigid benzene ring pieces and two freely rotating pyridyl arms combined by intervening methoxy, diazene, carbomethoxy groups. The N atoms of pyridyl arms are predisposed, as the primary group, to coordinate with metal ions for CPs formation, while leaving the neutral and generally weaker binding, methoxy, diazene, carbomethoxy groups as free-standing secondary donors to catch Cr(III). Aiming to search a CP-based sorbent with the sensitivity to Cr(III), therefore, reaction of L1 with Cd(OAc)_2_·2H_2_O and 1,4-benzenedicarboxylic acid in MeCN/H_2_O followed by a hydrothermal treatment at 170 °C for four days produced crystals of **1** with a 38% yield. In polymer **1**, although 1,4-benzenedicarboxylic acid does not coordinate in the products, its presence is crucial for the growth of crystalline products, and only poorly defined microcrystalline products can be obtained without it, which may be due to template effect of 1,4-benzenedicarboxylic acid. Moreover, it is interesting that the L1 ligand hydrolyzed and gave a 4-(2-(4-((pyridin-3-yl)methoxy)phenyl)diazenyl)benzoic acid (HL) in the hydrothermal process (Scheme 1). 

**Figure 1:**
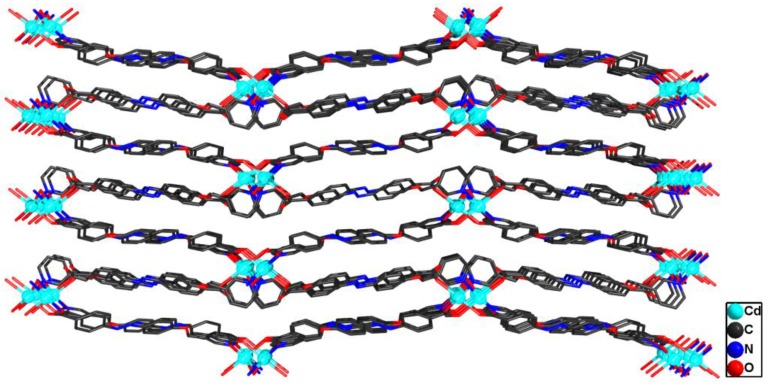
View of a 3D framework in **1** looking down the a axis.

Single-crystal X-ray diffraction analysis shows that polymer **1** crystallizes in the monoclinic space group *C*2/*c*. Its asymmetric unit contains one crystallographically independent Cd atom, two L^−^ ligands and a half coordinated water molecule. As shown in Figure S1a, Cd1 atom and its symmetry-related Cd1A are bridged by one H_2_O molecule and two carboxylate groups to form a dinuclear [Cd_2_(CO_2_)_2_(H_2_O)] unit, which can be used as secondary building unit (SBU). Each building unit is connected through carboxylate groups (μ_2_–η^1^:η^1^) and N atoms from L^−^ ligands to generate a 1D double-chain structure (Figure S1b). The neighboring 1D chains are linked via carboxylate groups (μ_1_–η^1^:η^0^) and N atoms from another L^−^ ligands to produce a 2D layer (Figure S1c). The 2D layers are further bridged by L^−^ ligands to afford a 3D framework (Figure 1). Topologically, the overall structure of **1** can be described as a four-connected 6^6^ topology (Figure S1d). Based on Figure 1, the diazene and methoxy groups freely stand in polymer **1**. Thus, polymer **1** would be a potential sorbent.

### 3.2. Cr(III) Adsorption Studies

**Figure 2:**
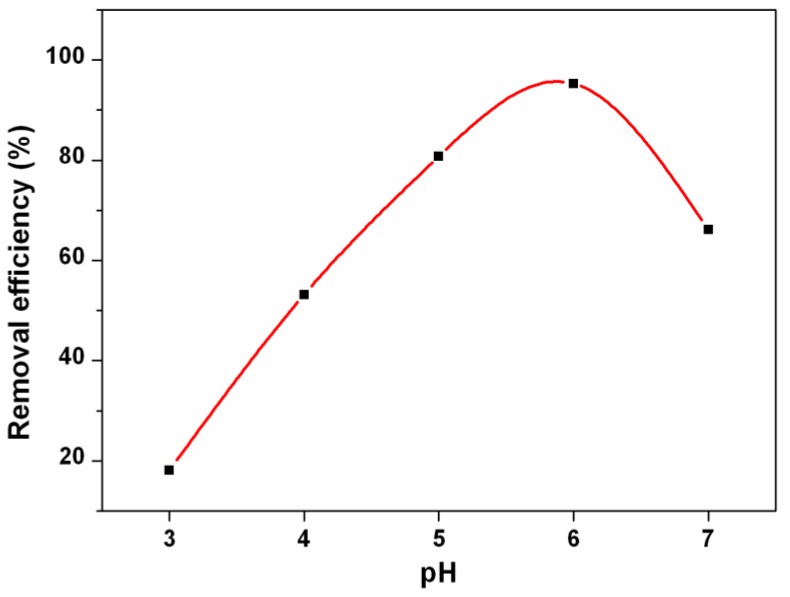
Effect of pH on the removal of Cr(III) by polymer **1**.

The pH of the sample solution has been proven to be an extremely important parameter for governing the removal of chromium species. In general, Cr(III) species varies over the pH value. It has been reported that Cr(III) exists mainly as [Cr(H_2_O)_6_]^3+^ at pH lower 4.0, [Cr(OH)]^2+^, [Cr(OH)_2_]^+^ at pH 4.0–7.0, and Cr(OH)_3_ at pH 7–10 Figure S5 [24,57,58,59]. The effect of pH on Cr(III) removal by polymer **1** was studied at different pH values ranging from 3.0 to 7.0 (Figure 2). The selection of this pH range was to avoid the hydroxide precipitation. As can be seen, removal efficiency of Cr(III) increased dramatically as the pH increased with the largest signal produced at pH 6.0. Further increase in the pH resulted in a decline in signal enhancement. Moreover, when the solution pH is below 4.0, the removal efficiency of **1** is very low, which is mainly due to the protonation of the diazene and methoxy groups and their diminishing ability to chelate Cr(III). When the solution pH is above 6.0, partial precipitation of hydroxide species of Cr(III) occurs. Thus, pH 6.0 of the Cr(III) solution was applied throughout the remaining work.

**Figure 3:**
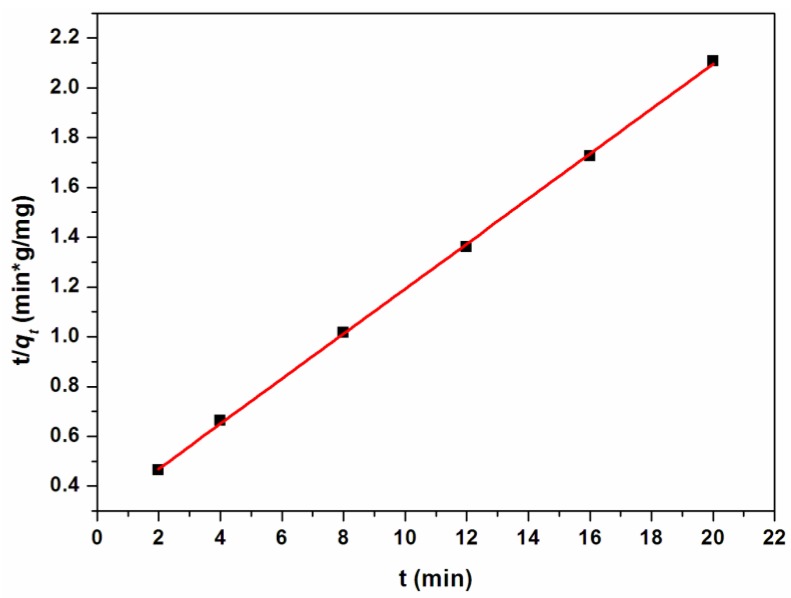
The pseudo-second-order kinetic plot for the adsorption.

In order to understand the Cr(III) adsorption kinetics of polymer **1**, the experimental data collected at the initial Cr(III) concentration of 1 ppm at pH 6 were fitted with the pseudo-second-order kinetic model with a high value of correlation coefficient (*R*^2^ = 0.9997) used to fit the kinetic results. The model can be expressed as:

(4) $$\frac{t}{q_{t}}=\frac{1}{k_{2}q_{e}^{2}}+\frac{t}{q_{e}}$$

where *k*_2_ (g·mg^−1^·min^−1^) is the kinetic rate constant for the pseudo-second-order model, *q*_e_ (mg·g^−1^) is the adsorption capacity at equilibrium, and *q*_t_ (mg·g^−1^) is the adsorption capacity at time *t* (min). The equilibrium adsorption capacity *q*_e_ and the pseudo-second-order rate *k*_2_ can be experimentally determined from the slope and the intercept of the plot *t*/*q*_t_ against *t* (Figure 3). The obtained rate constant, *k*_2_, was 0.028 g·mg^−1^·min^−1^ at 298 K. These results indicate that the pseudo-second-order mechanism is predominant, and chemisorptions may be the rate-limiting step that controls the adsorption process [51].

**Figure 4:**
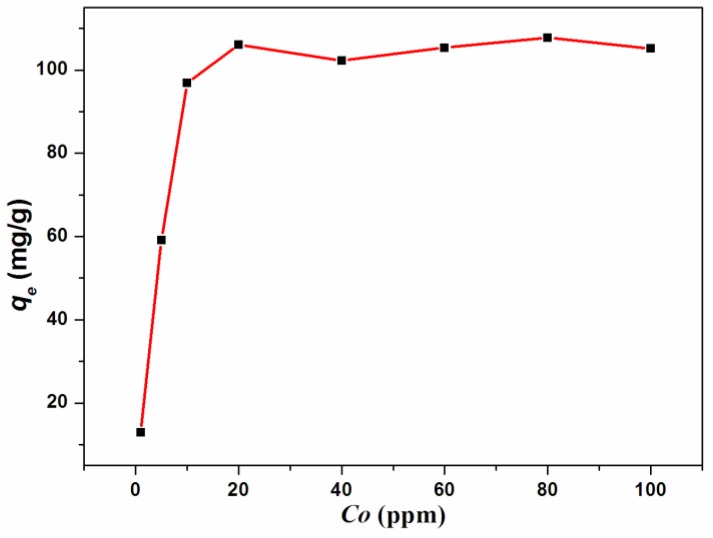
Adsorption curve of Cr(III) at different concentrations.

To evaluate the maximum sorption capacity and further understand the adsorbate–adsorbent interaction, the adsorption isotherm was measured at pH 6.0. As shown in Figure 4, the adsorption capacity increased with increasing initial concentrations and ultimately attained a saturated value. It is worth noting that polymer **1** shows a high Cr(III) uptake capacity of 106.13 mg·g^−1^, which is much higher than the inorganic absorbent (FePO_4_, 8.12 mg·g^−1^ [24] and vermiculite pure clay mineral, 46.948 mg·g^−1^ [60] or organic absorbent (3-(2-aminoethylamino)propyltrimethoxysilane, 30.5 mg·g^−1^) [23].

**Figure 5:**
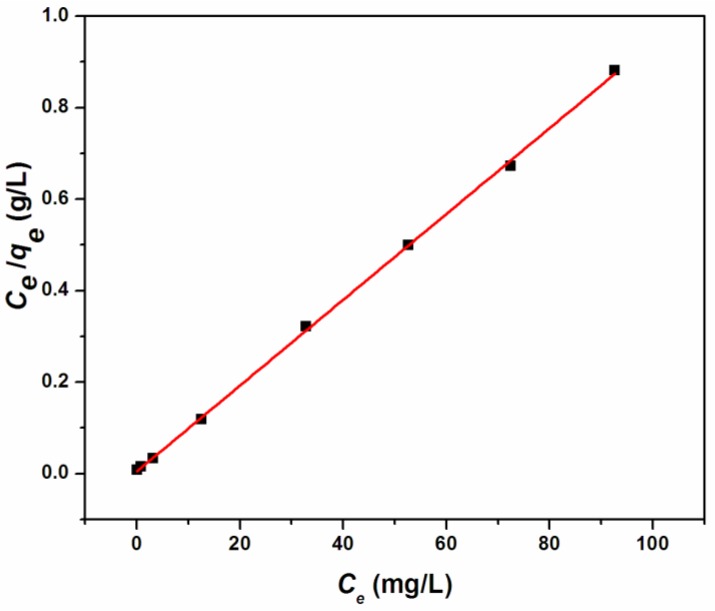
The linear regression by fitting the equilibrium adsorption data with the Langmuir adsorption model.

In order to derive an appropriate correlation between the experimental and theoretical data, the Langmuir model was applied (*R*^2^ = 0.9996), which is represented by Equation (5):

(5) $$\frac{C_{e}}{q_{e}}=\frac{C_{e}}{q_{m}}+\frac{1}{q_{m}K_{L}}$$

where *q*_m_ is the maximum amount of adsorption (mg·g^−1^), *q*_e_ is the adsorption capacity at equilibrium (mg·g^−1^), *C*_e_ is the equilibrium concentration of the remaining Cr(III) in the solution (mg·L^−1^), *K*_L_ is the Langmuir constant. The values of *q*_m_ and *K*_L_ were calculated from the slope and intercept of the *C*_e_/*q*_e_ vs *C*_e_ plot (Figure 5). The Langmuir isotherm is applicable to monolayer adsorption with all identical and energetically equivalent adsorption sites. The calculated maximum adsorption capacity *q*_m_ was obtained 106.61 mg·g^−1^, which was very close to the experimental data of 106.13 mg·g^−1^. 

**Figure 6:**
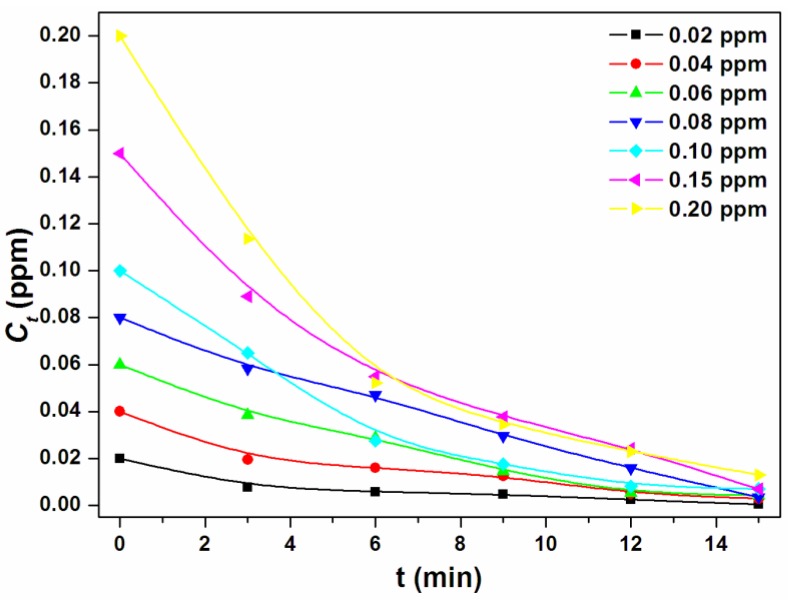
Adsorption kinetics of Cr(III) in aqueous solution by polymer **1**, using different initial concentrations of Cr(III) from 0.02 to 0.2 ppm.

To further investigate the removal efficiency for Cr(III), adsorption isotherms were collected from extremely dilute solutions ranging from 0.02 to 0.20 ppm under the optimized experimental conditions. As shown in Figure 6, fast kinetics and high removal efficiency were observed for polymer **1**, which can attain a removal efficiency higher than 92.5% in 15 min for various concentrations and is able to reduce the Cr(III) concentration of 0.1 ppm to the acceptable limit of 0.007 ppm for drinking water. By contrast, a reported post-synthetically modified CP material (UiO-66-NH_2_ decorated with thiourea, isothiocyanate and isocyanate groups) [19] shows effective Cr(III) removal in the 100 ppm magnitude of the Cr(III) concentration about 240 min-contact time, and the residual was more than 10 ppm, much higher than the criterion of the World Health Organization. This suggests that our material is an excellent adsorbent for Cr(III) removal, even in low-concentration magnitude.

**Figure 7:**
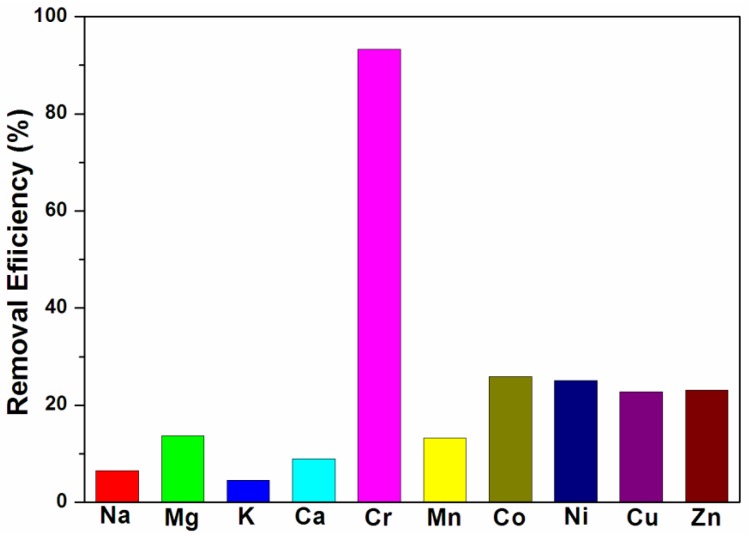
Adsorption kinetics of Cr(III) in aqueous solution by polymer **1**, using different initial concentrations of Cr(III) from 0.02 to 0.2 ppm.

The metal ions in the environment are often found to be in a matrix containing various ions. So, it is of great importance to investigate the selectivity for Cr(III) in the presence of coexisting ions. The selectivity of **1** toward Cr(III) was performed in a mixed solution containing Cr(III), Na(I), Mg(II), K(I), Ca(II), Mn(II), Co(II), Ni(II), Cu(II) and Zn(II). It is noteworthy that polymer **1** retains its high removal efficiency for Cr(III), even in the presence of background metal ions (Figure 7). To evaluate the binding properties of the adsorbent for different metal ions, distribution coefficients *K*_d_ was measured. The *K*_d_ represents an important aspect of any sorbent’s performance metrics of metal ion adsorption. In general, *K*_d_ values of ca. 10^3^ mL·g^−1^ are considered good, and those above 10^4^ mL·g^−1^ are outstanding [61]. The *K*_d_ of **1** for Cr(III) has been measured to be excellent with a value of 1.99 × 10^5^ mL·g^−1^, greatly higher than other metal ions (Table 1). These studies suggest that polymer **1** is an excellent adsorbent for select removal Cr(III) with high efficiency.

The specificity for Cr(III) removal may be ascribed to the coordination of Cr(III) with the functionalized methoxy and diazene groups of **1**. In other words, Cr(III) were adsorbed on the methoxy and diazene groups groups. It is widely acknowledged that the flexible geometry of Cr(III) results in a variety of coordination environments, which give a facile access to coordinate with Lewis base [35]. Furthermore, the highest ionic potential among the tested metal ions (Table S2) makes it more liable to display electrostatic adsorption [62] and effectively coordinate with **1,** leading to a higher adsorption capacity for Cr(III) than other metal ions. Interestingly, the ionic potential for Mg(II), Mn(II), Co(II), Ni(II), Cu(II), Zn(II) was obviously higher than Na(I), K(I), Ca(II), and the same result was also obtained for their removal efficiency, which further confirmed that higher ionic potential displays easier electrostatic adsorption.

### 3.3. The Stability of **1**

Polymer **1** was insoluble and stable in common organic solvents, such as chloroform, tetrahydrofuran and acetone. Importantly, it is also very stable in water. Meanwhile, thermogravimetric analysis (TGA) indicates that the framework of **1** is thermally stable up to 335 °C (Figure S2). To confirm the chemical stability of the polymer **1** after loading Cr(III), PXRD investigation was employed. As shown in Figure S3, the PXRD patterns of the polymer **1** after loading Cr(III) closely matches the as-synthesized samples, indicating the excellent chemical stability of the sorbent. 

### 3.4. The Mechanism for Removal of Cr(III)

**Figure 8:**
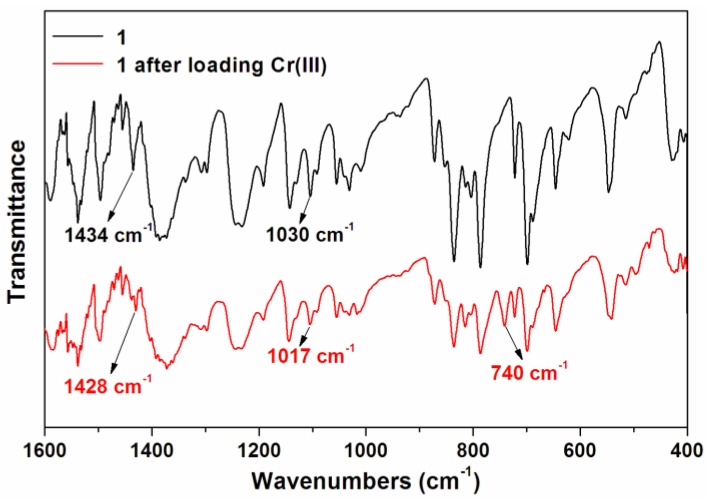
The infrared (IR) spectrum of as-synthesized samples and samples after loading Cr(III).

To realize the adsorption mechanism in this system, IR (Infrared Spectroscopy) was carried out for the as-synthesized samples and Cr(III) loaded samples (Figure 8). The appearance of a new peak at 740 cm^−1^ in the spectrum of samples after loading Cr(III) is typical of the characteristic stretching vibration of Cr–O [63], confirming the fact of Cr(III) loaded. For the as-synthesized samples, the peaks at 1434 and 1030 cm^−1^ are assigned to the characteristic group frequencies of the –N=N– and –O– vibrations, whereas these peaks are shifted to the 1428 and 1017 cm^−1^ for the CP materials after loading Cr(III). These obvious red shifts in the IR spectrum indicate the occurrence of complexation between Cr(III) and diazene/methoxy groups, because the coordination interaction could limit their stretching vibrations and consequently decrease their vibration frequency [64].

**Figure 9:**
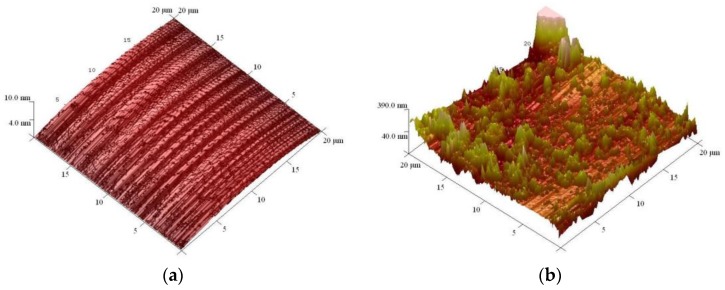
(**a**) Atomic force microscope (AFM) image of as-synthesized samples. (**b**) AFM image of samples after loading Cr(III).

**Figure 10:**
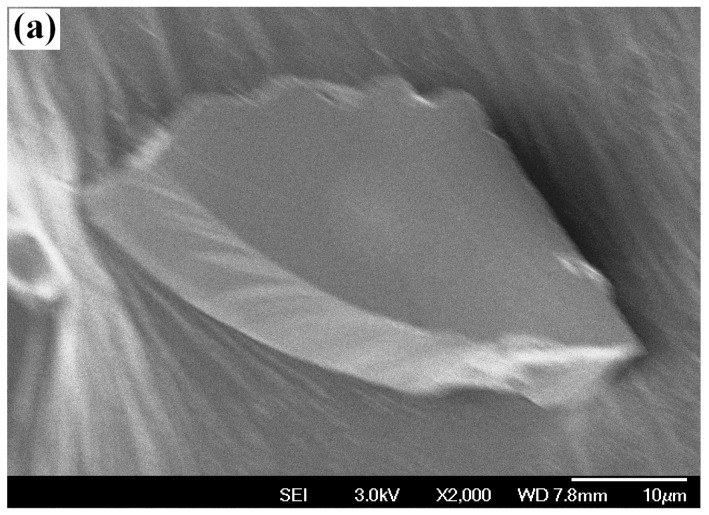

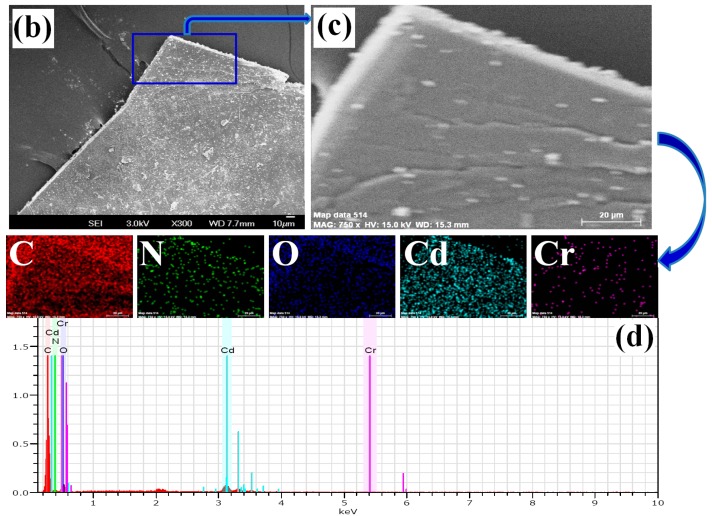
(**a**) Scanning electron microscope (SEM) image of as-synthesized samples. (**b**,**c**) SEM images of samples after loading Cr(III). (**d**) Energy dispersive spectrometer (EDS) spectra of the samples after loading Cr(III).

To gain further insights into the excellent performance for polymer **1** in the effective removal of Cr(III), which exhibits a high saturation uptake capacity and removal efficiency, AFM, SEM and energy dispersive spectrometer (EDS) measurements were conducted to evaluate the loaded Cr(III). As shown in Figure 9a, the AFM image of the as-synthesized samples displays very smooth surfaces, while the samples after loading Cr(III) exhibit many obvious light dots (Figure 9b), which confirmed that the loaded Cr(III) were adsorbed on the surfaces by the coordination between Cr(III) and diazene/methoxy groups. Furthermore, the SEM and EDS investigations were carried out to examine the surface morphology and chemical composition of the adsorbent after the adsorption of Cr(III). As illustrated in Figure 10a, the as-synthesized samples exhibit a crystalline phase with smooth surfaces, while many particles dotted over the surfaces of the samples treated by Cr(III) solution (Figure 10b,c). This result further confirmed that the Cr(III) species were adsorbed on the surfaces of samples, which is also verified by the EDS spectra (Figure 10d). Due to the diazene/methoxy groups populated not only on the surface but also in the pore of the adsorbent, we inferred that adsorption may happen in the inner surface of the adsorbent; so, a cross-section of EDS measurement was performed. The spectra also showed the existence of Cr(III) (Figure S4), implying that the Cr(III) uptake occurred inside adsorbents, which was also observed in the Zn(II)-based metal–organic framework for removal of Cd(II) [39]. These results further confirmed that the densely populated free standing methoxy and diazene groups, which located on the surface and inner pores of the CP, could effectively capture the Cr(III), resulting in a large adsorption capacity and ultra-low residual concentration, even in relatively low concentrations.

## 4. Conclusions

In summary, we have presented the synthesis and structural characterization of a novel CP decorated with the diazene and methoxy groups, for effective and specific removal of Cr(III). Notably, without any pre-treatment, polymer **1** not only exhibits a high Cr(III) uptake capacity (106.13 mg·g^−1^), but also displays a good efficiency towards the removal Cr(III) from aqueous solution, even for the trace Cr(III) (0.02–0.20 ppm). The characterization results for CP before and after adsorption of Cr(III) suggest that the good performance for Cr(III) removal by CP mainly stem from capture feature of diazene and methoxy groups, which happened not only on the surfaces of the adsorbent, but also in the pores of CP, and thus could efficiently remove Cr(III). This offers a new way to design and synthesize CPs decorated with free standing groups as a platform for removing other heavy metal ions from aqueous solution.

## Figures and Tables

**Table 1:** Distribution coefficients of various metal ions by polymer **1**.

Metal ions	Cr^3+^	Na^+^	Mg^2+^	K^+^	Ca^2+^	Mn^2+^	Co^2+^	Ni^2+^	Cu^2+^	Zn^2+^
*K*_d_ (mL·g^−1^)	199,253	990	2262	673	1401	2172	4980	4777	4202	4294

## Supplementary Materials

The following are available online at www.mdpi.com/ 2073-4360/9/7/273/s1, Figure S1: (a) View of dinuclear [Cd_2_(CO_2_)_2_(H_2_O)] unit of 1. (b) View of a 1D double chain in 1 extending along the ac plane. (c) View of a 2D layer in 1 extending along the bc plane. (d) Schematic view of the 4-connected 66 topology of 1. Atom color codes: Cd, cyan; O, red; N, blue; C, dark green and pink, Figure S2: TG curve coupled with IR spectra of 1, Figure S3: The PXRD patterns from simulated single crystal data, of as-synthesized samples, of as-synthesized samples after immerging in chromium solution, Figure S4: (a,b) SEM images of the section for samples after loading Cr(III). (c) EDS spectra of the section for samples after loading Cr(III); Figure S5: Effect of pH on the Cr(III) distribution in aqueous solution (1 ppm), Table S1: Summary of Crystallographic Data for **1**, Table S2: Comparison of Ionic Potential.
