# Supplementary material for: Effective Removal of Chromium(III) from Low Concentration Aqueous Solution Using a Novel Diazene/Methoxy-Laced Coordination Polymer
Source: Polymers (Basel). 2017 Jul 9;9(7):273. doi: 10.3390/polym9070273 (PMC6431845; doi:10.3390/polym9070273)

checkCIF/PLATON report

You have not supplied any structure factors. As a result the full set of tests cannot be run.

THIS REPORT IS FOR GUIDANCE ONLY. IF USED AS PART OF A REVIEW PROCEDURE FOR PUBLICATION, IT SHOULD NOT REPLACE THE EXPERTISE OF AN EXPERIENCED CRYSTALLOGRAPHIC REFEREE.

No syntax errors found. CIF dictionary Interpreting this report

Datablock: t

```
Bond precision:  C-C = 0.0053 A          Wavelength=0.71073

Cell:             a=12.430(3)     b=15.186(3)       c=36.429(7)
                  alpha=90        beta=97.23(3)     gamma=90
Temperature:      296 K

                Calculated                 Reported
Volume          6822(3)                    6822(2)
Space group     C 2/c                      C 2/c
Hall group      -C 2yc                     C 2yc
Moiety formula  C76 H58 Cd2 N12 O13        C76 H58 Cd2 N12 O13
Sum formula     C76 H58 Cd2 N12 O13        C76 H58 Cd2 N12 O13
Mr              1572.16                    1572.14
Dx,g cm-3       1.531                      1.531
Z               4                          4
Mu (mm-1)       0.699                      0.699
F000            3192.0                     3192.0
F000'           3186.85
h,k,lmax        14,18,43                   14,18,43
Nref            6007                       6001
Tmin,Tmax       0.870,0.932                0.873,0.933
Tmin'           0.870

Correction method= # Reported T Limits: Tmin=0.873 Tmax=0.933
AbsCorr = MULTI-SCAN

Data completeness= 0.999          Theta(max)= 25.000

R(reflections)= 0.0364( 4471)     wR2(reflections)= 0.0857( 6001)

S = 1.022                  Npar= 465
```

The following ALERTS were generated. Each ALERT has the format
test-name_ALERT_alert-type_alert-level.
Click on the hyperlinks for more details of the test.

Alert level C

```
PLAT127_ALERT_1_C Implicit Hall Symbol  Inconsistent with Explicit      C 2yc
PLAT230_ALERT_2_C Hirshfeld Test Diff for    N2     --  C8      ..        5.7 s.u.
PLAT241_ALERT_2_C High   'MainMol' Ueq as Compared to Neighbors of        C17 Check
PLAT241_ALERT_2_C High   'MainMol' Ueq as Compared to Neighbors of        C36 Check
PLAT242_ALERT_2_C Low    'MainMol' Ueq as Compared to Neighbors of         N6 Check
PLAT242_ALERT_2_C Low    'MainMol' Ueq as Compared to Neighbors of        C15 Check
PLAT242_ALERT_2_C Low    'MainMol' Ueq as Compared to Neighbors of        C34 Check
PLAT250_ALERT_2_C Large U3/U1 Ratio for Average U(i,j) Tensor ....        2.3 Note
```

Alert level G

```
PLAT004_ALERT_5_G Polymeric Structure Found with Maximum Dimension          3 Info
PLAT005_ALERT_5_G No Embedded Refinement Details found in the CIF      Please Do !
PLAT007_ALERT_5_G Number of Unrefined Donor-H Atoms ..............          1 Report
PLAT066_ALERT_1_G Predicted and Reported Tmin&Tmax Range Identical          ? Check
PLAT083_ALERT_2_G SHELXL Second Parameter in WGHT  Unusually Large      11.23 Why ?
PLAT710_ALERT_4_G Delete 1-2-3 or 2-3-4 Linear Torsion Angle ... #         88 Do !
            O4  -CD1 -O1  -C1    -88.50  1.70   1.555   1.555   1.555   1.555
PLAT710_ALERT_4_G Delete 1-2-3 or 2-3-4 Linear Torsion Angle ... #         97 Do !
            O1  -CD1 -O4  -C20    54.00  1.80   1.555   1.555   1.555   1.555
PLAT804_ALERT_5_G Number of ARU-Code Packing Problem(s) in PLATON           1 Info
PLAT899_ALERT_4_G SHELXL97   is Deprecated and Succeeded by SHELXL       2014 Note
```

0 **ALERT level A** = Most likely a serious problem - resolve or explain
0 **ALERT level B** = A potentially serious problem, consider carefully
8 **ALERT level C** = Check. Ensure it is not caused by an omission or oversight
9 **ALERT level G** = General information/check it is not something unexpected

2 ALERT type 1 CIF construction/syntax error, inconsistent or missing data
8 ALERT type 2 Indicator that the structure model may be wrong or deficient
0 ALERT type 3 Indicator that the structure quality may be low
3 ALERT type 4 Improvement, methodology, query or suggestion
4 ALERT type 5 Informative message, check

It is advisable to attempt to resolve as many as possible of the alerts in all categories. Often the minor alerts point to easily fixed oversights, errors and omissions in your CIF or refinement strategy, so attention to these fine details can be worthwhile. In order to resolve some of the more serious problems it may be necessary to carry out additional measurements or structure refinements. However, the purpose of your study may justify the reported deviations and the more serious of these should normally be commented upon in the discussion or experimental section of a paper or in the "special_details" fields of the CIF. checkCIF was carefully designed to identify outliers and unusual parameters, but every test has its limitations and alerts that are not important in a particular case may appear. Conversely, the absence of alerts does not guarantee there are no aspects of the results needing attention. It is up to the individual to critically assess their own results and, if necessary, seek expert advice.

Publication of your CIF in IUCr journals

A basic structural check has been run on your CIF. These basic checks will be run on all CIFs submitted for publication in IUCr journals (*Acta Crystallographica*, *Journal of Applied Crystallography*, *Journal of Synchrotron Radiation*); however, if you intend to submit to *Acta Crystallographica Section C* or *E*, you should make sure that full publication checks are run on the final version of your CIF prior to submission.

Publication of your CIF in other journals

Please refer to the *Notes for Authors* of the relevant journal for any special instructions relating to CIF submission.

PLATON version of 19/11/2015; check.def file version of 17/11/2015

Datablock t - ellipsoid plot

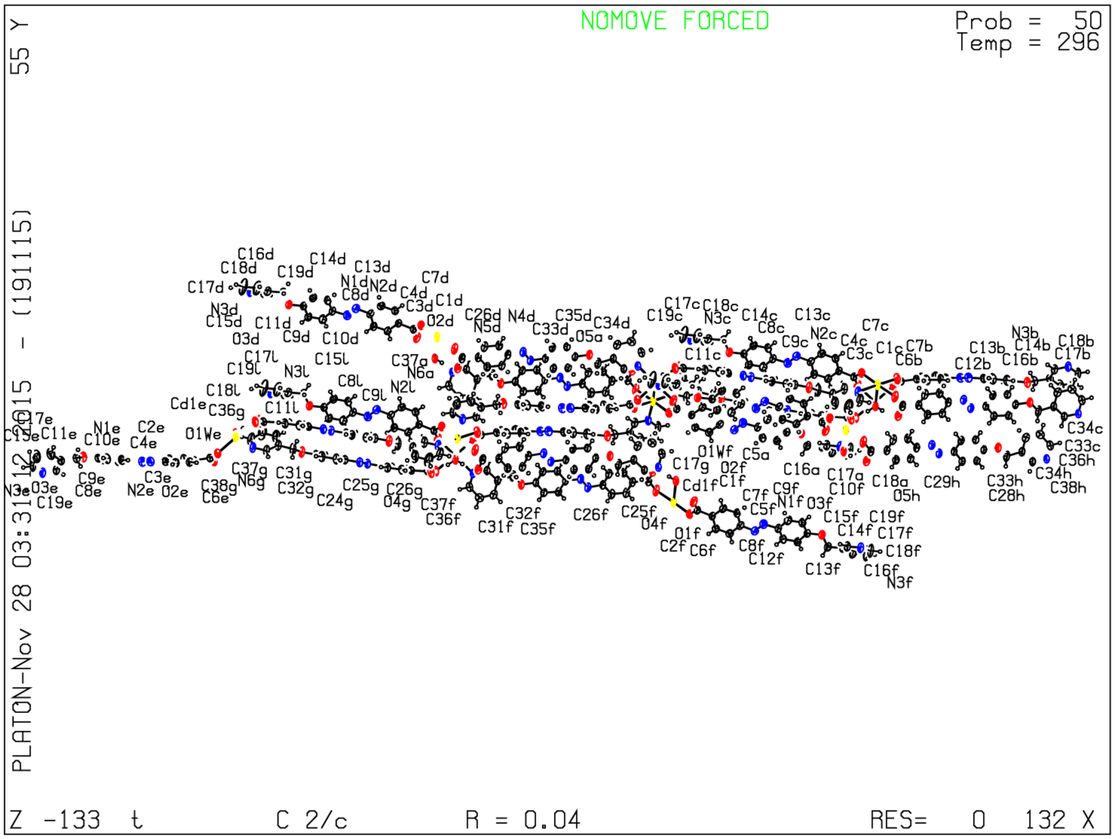

Supplement: Supplementary file 1 [file polymers-09-00273-s001.zip › The check report for the single crystal data of 1.pdf]
