# Supplementary material for: Effective Removal of Chromium(III) from Low Concentration Aqueous Solution Using a Novel Diazene/Methoxy-Laced Coordination Polymer
Source: Polymers (Basel). 2017 Jul 9;9(7):273. doi: 10.3390/polym9070273 (PMC6431845; doi:10.3390/polym9070273)
Supplement: Supplementary file 1 [file polymers-09-00273-s001.zip › Supplementary Information.pdf]

polymers

MDPI

Article

Effective Removal of Chromium(III) from Low Concentration Aqueous Solution Using a Novel Diazene/Methoxy-Laced Coordination Polymer

Lei-Lei Liu *, Yun Xing, Hui-Ying Yu, Cai-Wen Zhang, Meng-Qi Ye, Ming-Zhen Miao and Cai-Xia Yu *

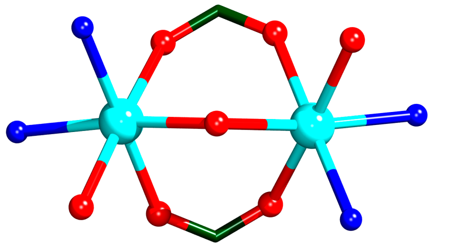

(**a**)

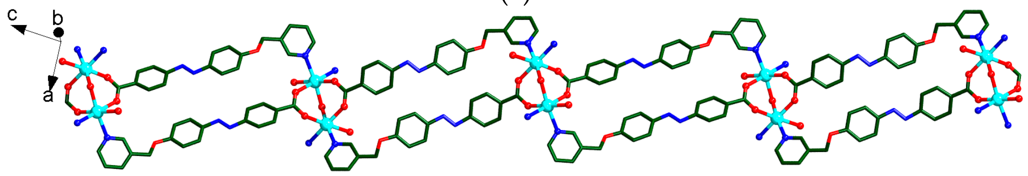

(**b**)

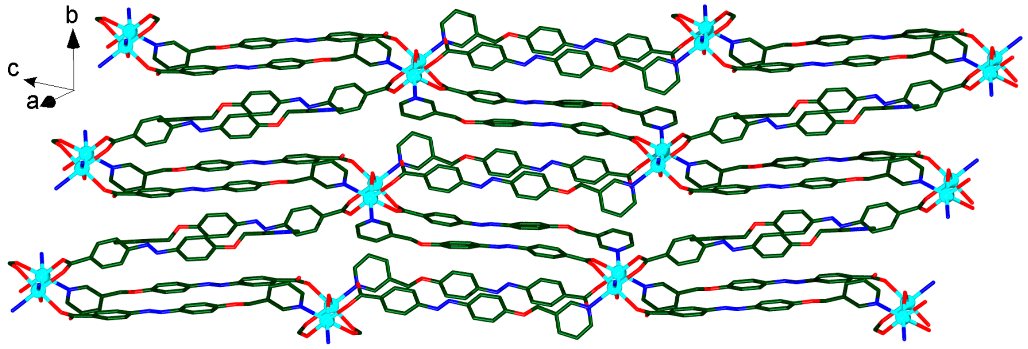

(**c**)

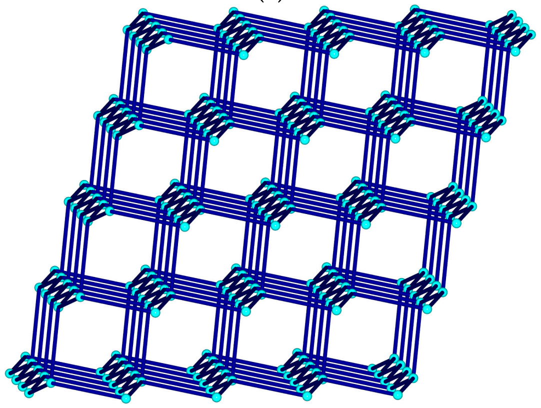

(**d**)

Figure S1. (**a**) View of dinuclear $[Cd_2(CO_2)_2(H_2O)]$ unit of **1**. (**b**) View of a 1D double chain in **1** extending along the *ac* plane. (**c**) View of a 2D layer in **1** extending along the *bc* plane. (**d**) Schematic view of the 4-connected 6^6 topology of **1**. Atom color codes: Cd, cyan; O, red; N, blue; C, dark green and pink.

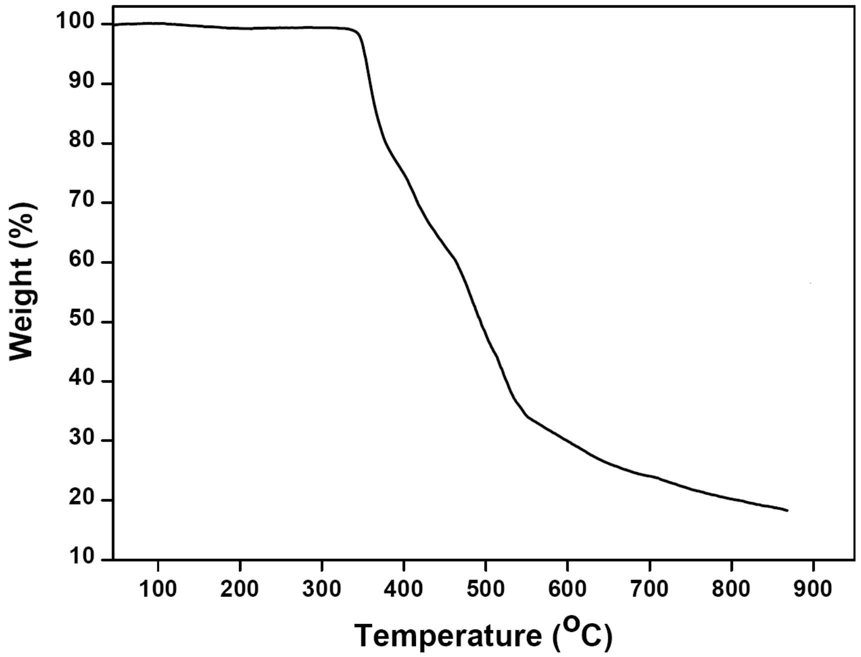

Figure S2. TG curve coupled with IR spectra of **1**.

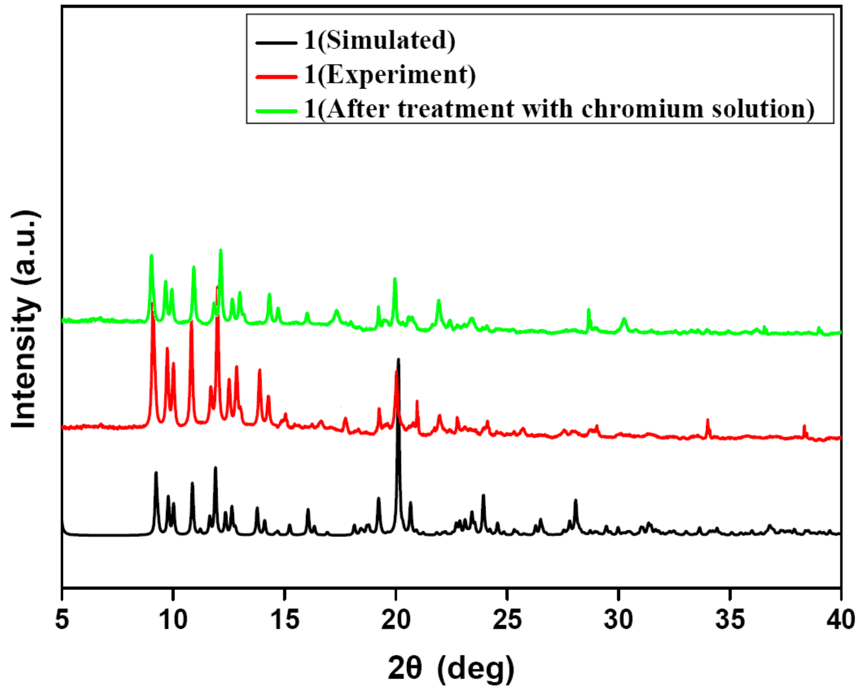

Figure S3. The PXRD patterns from simulated single crystal data, of as-synthesized samples, of as-synthesized samples after immerging in chromium solution.

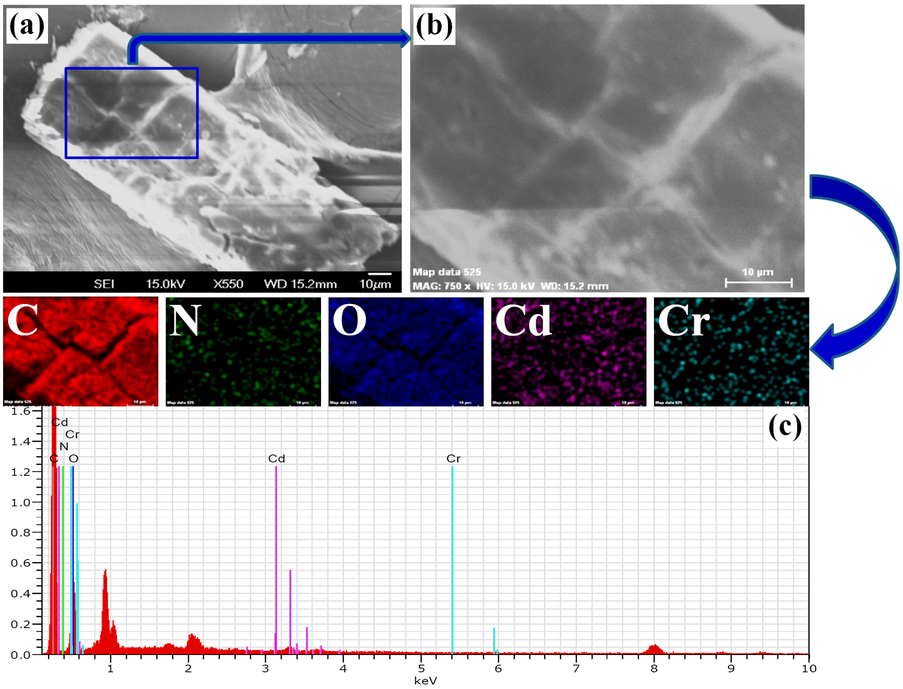

Figure S4. (**a**–**b**) SEM images of the section for samples after loading Cr(III). (**c**) EDS spectra of the section for samples after loading Cr(III).

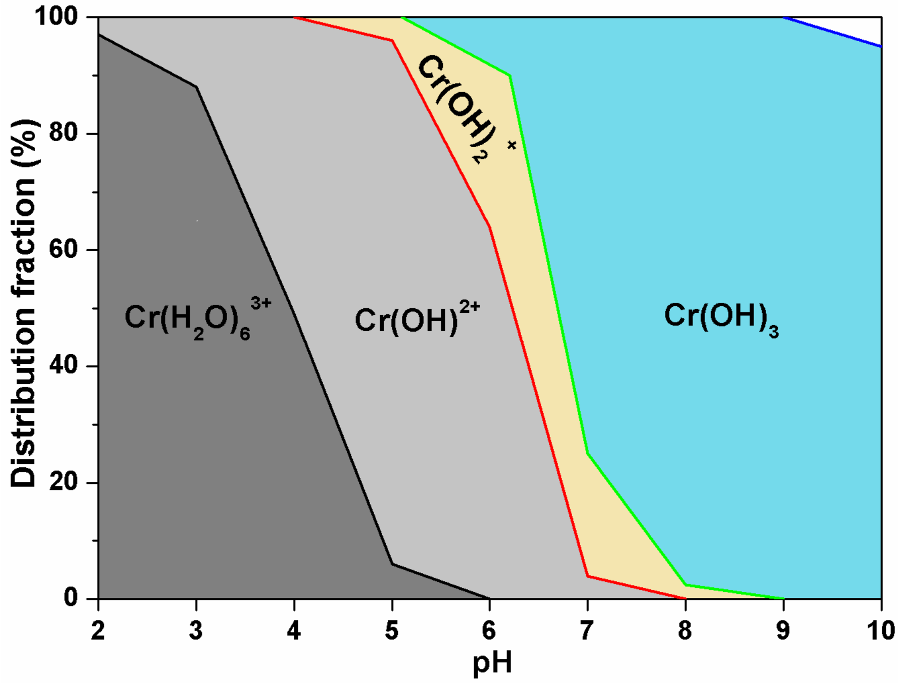

Figure S5. Effect of pH on the Cr(III) distribution in aqueous solution (1 ppm).

Table S1. Summary of Crystallographic Data for **1**.

Empirical Formula	$C_{76}H_{58}Cd_2N_{12}O_{13}$
Formula Weight	1572.14
Crystal System	monoclinic
Space Group	*C*2/*c*
a (Å)	12.430(3)
b (Å)	15.186(3)
c (Å)	36.429(7)
α (°)	90
β (°)	97.23(3)
γ (°)	90
V (Å^3)	6822(2)
Z	4
T (K)	296(2)
ρ_{calc} (g/cm^3)	1.531
F(000)	3192
μ (MoKα, mm^{-1})	0.699
Total reflections	25933
Unique reflections	6001 (R_{int} = 0.0459)
No. of observations	4471
No. of parameters	465
R_1 [a]	0.0364
wR_2 [b]	0.0857
GOF [c]	1.022

[a] $R_1 = \Sigma||F_o|-|F_c|/\Sigma|F_o|$. [b] $wR_2 = \{\Sigma w(F_o^2-F_c^2)^2/\Sigma w(F_o^2)^2\}^{1/2}$. [c] GOF = $\{\Sigma w(F_o^2-F_c^2)^2/(n-p)\}^{1/2}$, where *n* = number of reflections and *p* = total numbers of parameters refined.

Table S2. Comparison of Ionic Potential.

Metal ions	Na^{+}	Mg^{2+}	K^{+}	Ca^{2+}	Cr^{3+}	Mn^{2+}	Co^{2+}	Ni^{2+}	Cu^{2+}	Zn^{2+}
Ionic Radius (pm)	102	72	138	100	62	67	65	69	73	74
Ionic Potential	0.0098	0.0278	0.0072	0.0200	0.0484	0.0299	0.0308	0.0290	0.0274	0.0270
